# Data-driven strategies for the computational design of enzyme thermal stability: trends, perspectives, and prospects: Data-driven strategies for enzyme thermostability design

**DOI:** 10.3724/abbs.2023033

**Published:** 2023-03-16

**Authors:** Zhixin Dou, Yuqing Sun, Xukai Jiang, Xiuyun Wu, Yingjie Li, Bin Gong, Lushan Wang

**Affiliations:** 1 State Key Laboratory of Microbial Technology Shandong University Qingdao 266237 China; 2 School of Software Shandong University Jinan 250101 China; 3 National Glycoengineering Research Center Shandong University Qingdao 266237 China

**Keywords:** enzyme design, data-driven, machine learning, thermal stability

## Abstract

Thermal stability is one of the most important properties of enzymes, which sustains life and determines the potential for the industrial application of biocatalysts. Although traditional methods such as directed evolution and classical rational design contribute greatly to this field, the enormous sequence space of proteins implies costly and arduous experiments. The developm ent of enzyme engineering focuses on automated and efficient strategies because of the breakthrough of high-throughput DNA sequencing and machine learning models. In this review, we propose a data-driven architecture for enzyme thermostability engineering and summarize some widely adopted datasets, as well as machine learning-driven approaches for designing the thermal stability of enzymes. In addition, we present a series of existing challenges while applying machine learning in enzyme thermostability design, such as the data dilemma, model training, and use of the proposed models. Additionally, a few promising directions for enhancing the performance of the models are discussed. We anticipate that the efficient incorporation of machine learning can provide more insights and solutions for the design of enzyme thermostability in the coming years.

## Introduction

Enzymes that have evolved over 3.5 billion years accelerate chemical reactions for the maintenance of life, adapting to a range of approximately ‒20°C to 120°C [ 1– 4] . The variation in catalytic activity exhibited by different temperature-adapted enzymes demonstrates the complex and critical temperature dependence of biocatalysts [5]. Hence, thermal stability serves as one of the most important factors for the efficient catalytic function of enzymes. Enzymes are considered highly efficient, versatile biocatalysts widely involved in a variety of industrial applications, including food, beverages, biorefineries, pharmaceuticals, and the degradation of toxic environmental pollutants [ 6– 10] . Nevertheless, in practical applications, a number of enzyme catalysis reactions must be performed at extreme temperatures [ 11– 15] . Thus, the thermal stability of enzymes is the main bottleneck in the industrial application of biocatalysts.

Enzyme thermal stability refers to the range of temperatures at which the enzymes can remain thermodynamically stable for catalytic function. For example, the rigid backbone of the 3D structure of hyperthermophilic enzymes can maintain the active and stable conformation to resist irreversible denaturation at extremely high temperatures [16]. Moreover, both thermal stability and thermodynamic stability should be incorporated into the workflow of enzyme design, avoiding misfolding or destabilized conformation of designed target proteins [ 17– 19] .

For decades, early research by enzyme engineering groups focused on improving activity or designing new functions using directed evolution and classical rational design methods [ 20– 26] . However, costly experiments and complicated factors related to protein thermal stability have led to difficulties and limitations in enzyme engineering [ 27– 32] . In 1973, Anfinsen proposed that the protein primary sequence determines the tertiary structure, greatly inspiring the field of protein design [33]. However, the precise and efficient design of enzymes is challenging due to the complex discontinuous mapping relationships among protein sequence, structure, and function. In terms of sequence complexity, for example, a protein of 100 amino acids in length has a sequence space of 20 ^100^ (~10 ^130^), which is well beyond the order of magnitude of proteins that have been sampled in nature (~10 ^12^) [34]. Such an immense protein sequence space has far exceeded the workload that can be covered by directed evolution or traditionally rational design methods [35]. Therefore, significantly reducing the scale of protein sequence space is highly important for efficient and accurate enzyme engineering.

**Figure 1:**
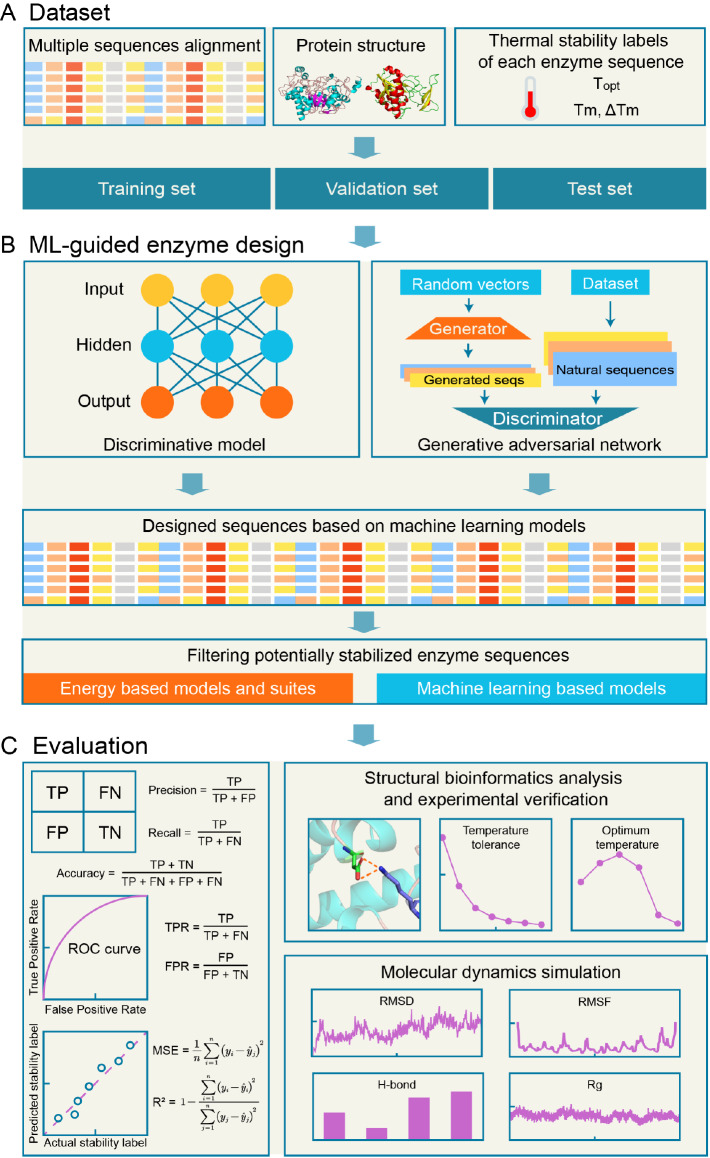
Figure 1 Architecture of thermal stability design for enzymes based on machine learning (A) The model can be trained on diverse datasets, including multiple sequence alignments, protein structures, and enzyme thermal stability labels. Furthermore, data splitting is a typical technical strategy to develop and evaluate machine learning models to improve the robustness and accuracy of the models. Specifically, the training dataset is used to fit the parameters to the machine learning models. Hyperparameters are combined and fine-tuned based on the validation set to improve the generalization performance of the model. Eventually, the test set is responsible for the unbiased evaluation of the machine learning models. (B) Two types of supervised machine learning algorithms provide potentially viable solutions for designing enzyme thermal stability. A large number of existing machine learning models effectively incorporate discriminative models and generative adversarial networks to guide protein engineering [ 43– 47] . Energy-based and machine learning–based methods help filter thermodynamically stable protein sequences. (C) As shown on the left, a variety of metrics are commonly leveraged to evaluate the performance of discriminative models. However, the validation results of biological analysis and experiments govern the validity of the models in enzyme engineering. Apart from the aforementioned methods, the preliminary screening and verification of candidates can be performed through molecular dynamics simulation technology [48]. C is modified from ref. [49].

The development of next-generation sequencing has had a significant impact on data-intensive studies in biology. In the data-driven research paradigm, machine learning-based methods for enzyme design have received much attention in recent years [ 36– 38] . Here, we propose an architecture for the thermal stability design of enzymes based on supervised machine learning models ( Figure 1). Compared with traditional enzyme engineering methods, intelligent design strategies can notably improve the efficiency and reduce the experimental cost for enzyme engineering, which has become a promising trend in the context of the upcoming fourth industrial technology revolution [ 39– 42] .

## Overview of Machine Learning

Machine learning relies on analyzing and learning patterns from datasets to update the model parameters for predicting new samples. Additionally, the methods can often provide a reusable and efficient solution when the volume of data or multidimensional complex features is too large to analyze manually or when an automated analysis is desired [50]. In this review, we introduce some basic concepts about traditional machine learning and deep neural networks.

### Traditional machine learning models

**Figure 2:**
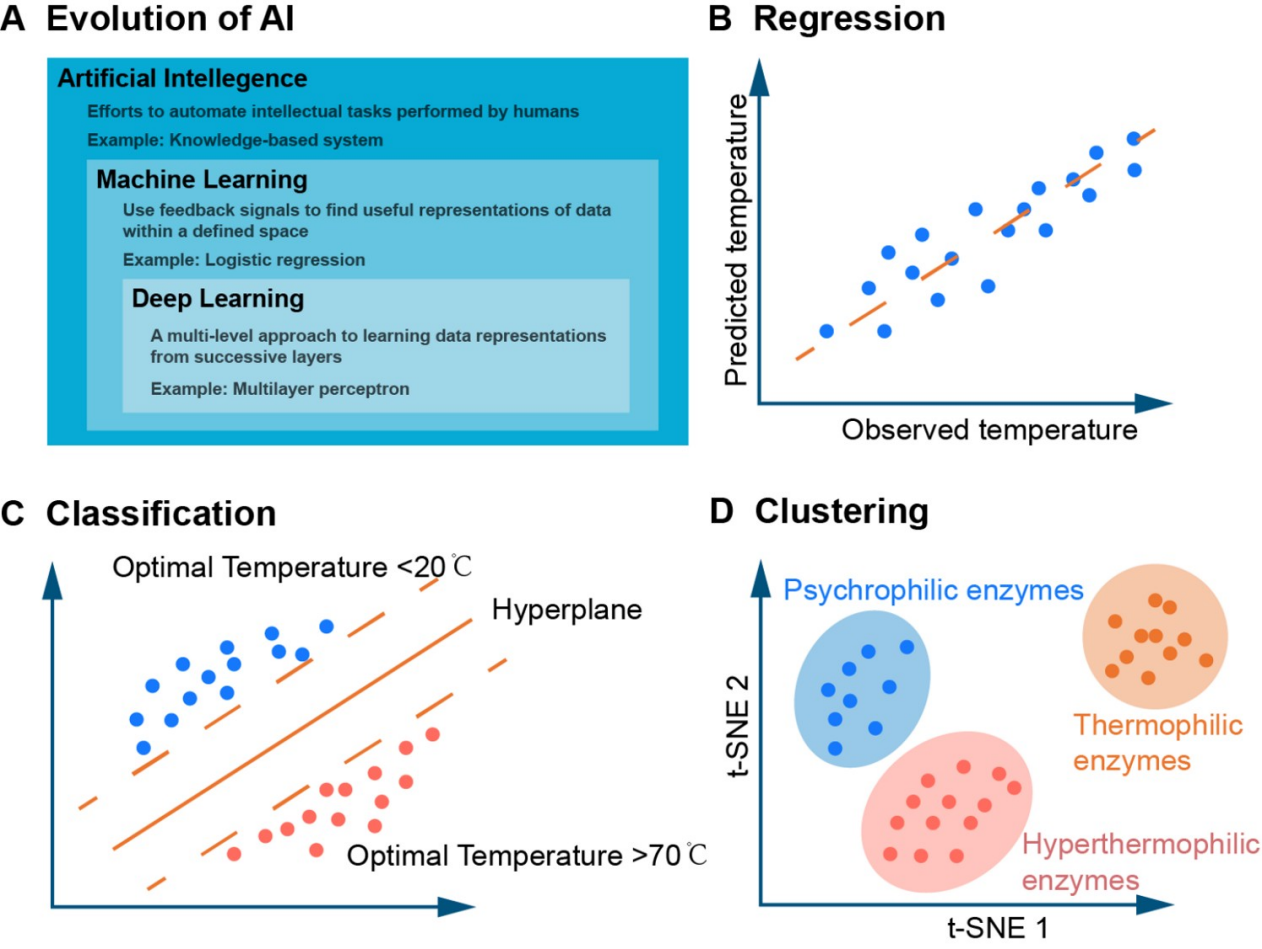
Figure 2 Machine learning-related terms and methods (A) Relationship between artificial intelligence, machine learning, and deep learning is illustrated with typical examples. (B) Regression models build a function describing a mathematical relationship between an independent variable (observed temperature) and a dependent variable (predicted temperature) [51]. (C) Classification models, such as support vector machines (SVMs), transform two groups of data in a distinct way as much as possible [52]. (D) Clustering model groups objects with similar characteristics into different clusters. For example, the different optimal temperatures of enzymes can be grouped using multiple sequence alignment (MSA) features [53]. B, C and D are modified from ref. [50].

As an important branch of artificial intelligence (AI), traditional machine learning methods are widely used because of the high interpretability of algorithms and low training costs. Thus, these models are still the primary choice when dealing with emerging disciplines and small-volume datasets ( Figure 2).

### Deep neural networks

**Figure 3:**
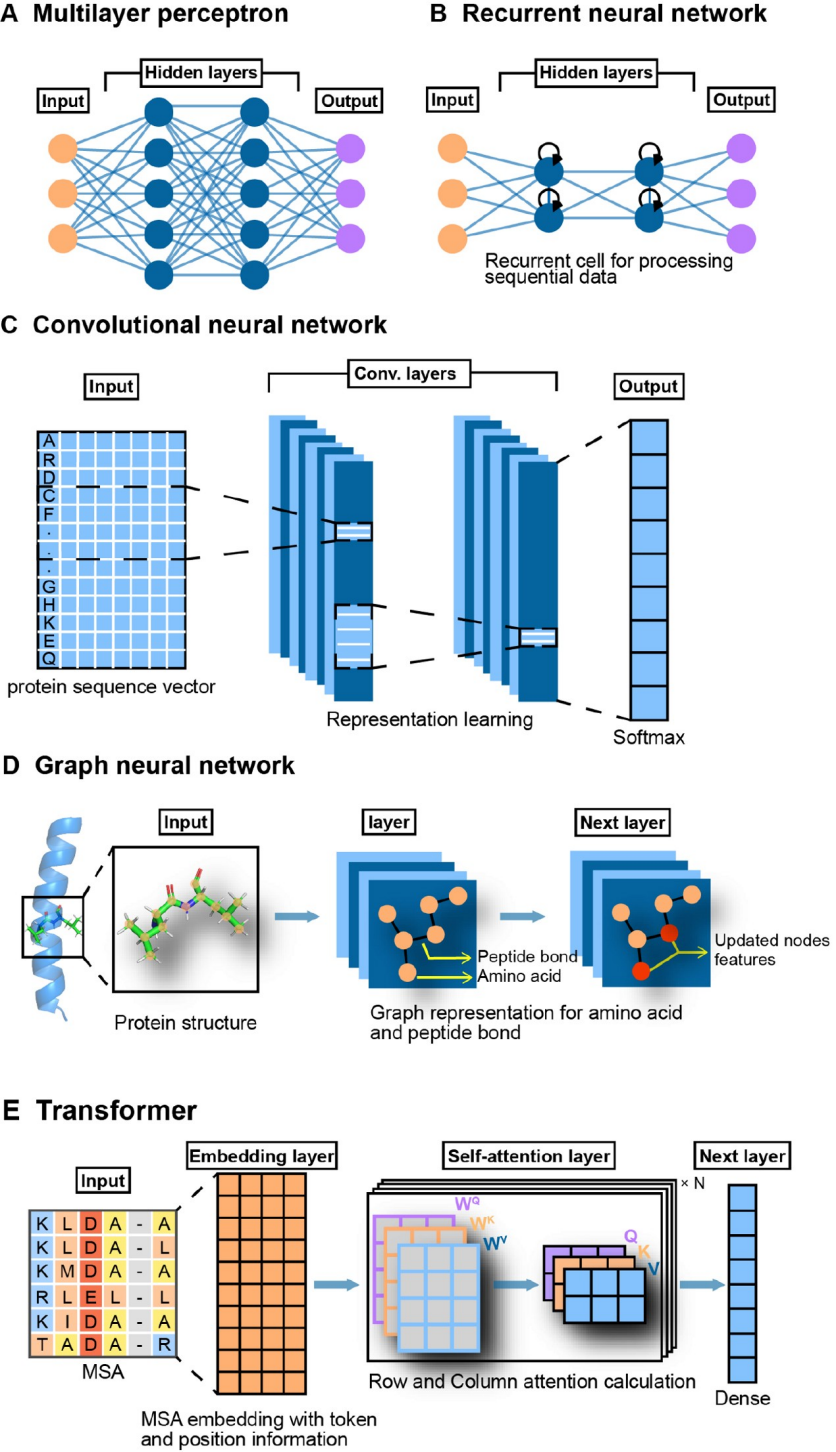
Figure 3 Neural network methods (A) A multilayer perceptron (MLP) is a class of fully connected neural network models including the input layer, the hidden layer, and the output layer [61]. In MLP, the neurons (nodes) process information by non-linear activation function [62]. (B) A recurrent neural network (RNN) takes input sequence elements and saves them as a hidden state that contains information related to the previous content. Data such as protein sequences have a sequential order that needs to be followed strictly to carry genetic information. Thus, RNNs are powerful models for processing biological sequence data [63]. (C) Convolutional neural networks (CNNs) use convolutional kernels to perform mathematical operations called convolution, which learn the spatial hierarchy of training data [64]. (D) Graph neural networks (GNNs) are a useful algorithm for leveraging graph-structured knowledge. Biological information, such as protein structure, can be described as a topological relationship among atoms and bonds [65]. (E) As an encoder–decoder architecture, the key components of Transformer consist of a multi-headed self-attention layer, followed by a feedforward layer [66]. Additionally, the performance of Transformer is heavily dependent on the first layer, which is the multi-headed self-attention layer [67]. Vector K, vector Q, and vector V are three different representations for inputs, which are calculated from the corresponding weight matrix to predict the importance of other segments to the current segment. Recently, Transformer has also been found to be potent when dealing with protein sequences and learning the relationships of residues such as interactions within proteins [ 68– 70] .

In the 1940s, McCulloch and Pitts first proposed a computational model of neurons, opening up the field of algorithmic research in deep learning [54]. In 1989, the universal approximation theorem proposed by Hornik *et al*. [55] provided a mathematical proof of deep learning and became one of the important theoretical bases for deep learning algorithms. In the last few years, a variety of different deep learning models have made outstanding contributions to life science [ 56– 59] ( Figure 3). Notably, AlphaFold2 uses MSA Transformer architecture to achieve an unprecedented level of accuracy in the end-to-end prediction of protein 3D structure [60].

A large number of open-source toolkits and frameworks are available to facilitate the use of AI technologies [ 71– 82] . The advantage of deep learning over traditional machine learning algorithms is that it automates the learning of features from datasets and eliminates the need for manual feature engineering. Therefore, the size and quality of the datasets intrinsically become some of the dominant factors for deep neural networks.

## Datasets Related to the Thermal Stability of Enzymes

Using machine learning to guide the design of the thermal stability of enzymes is inherently complex and challenging. The data-driven approaches to solving this problem, in turn, are limited by high-quality and large-scale datasets. As a result, multiple groups have worked extensively on dataset collection and refinement ( Table 1). We classified them into two categories for different methods of data collection: (1) large-scale data predicted by predictive models and (2) high-quality data collected from the published literature, released datasets, and high-throughput experiments. Some pros and cons are also presented in Table 1.

**Table:** **Table 1** Enzyme thermal stability datasets

Dataset description	Collection method	Dataset scale	Advantage	Disadvantage	Availability	Ref.
Tome: Optimal temperature of enzyme	Predicted by linear models, Bayesian ridge, and support vector regression	4447 enzyme families, 6,500,000 sequences	Large-scale protein sequences and family diversity facilitating training of machine learning models; easy-to-access public dataset	Insufficient accuracy of prediction methods; low accuracy of data on the optimum temperature of enzymes in the extreme temperature range	https://zenodo.org/record/2539114	[51]
The optimal growth temperature of bacteria and the optimal temperature of enzyme	Correlation analysis between enzyme temperature optima and the organism growth temperatures	21,498 OGT of bacteria	UniProt sequences covered by 43%; easy-to-access public dataset; covering a variety of temperature-adapted bacteria and archaea.	Stricter quality control needed; not actively maintained	https://doi.org/10.5281/zenodo.1175608	[83]
BRENDA: The optimal temperature of enzymes	Published papers in PubMed	32,000,000 sequences with 41,000 optimal temperature labels	High-quality data collected from the published literature; actively maintained; convenient web interface	Relatively small proportion of temperature-labeled sequences in protein families; no obviously experimental conditions	https://www.brenda-enzymes.org/	[84]
ThermoMutDB: Environment, ΔΔ *G*, Δ *T*_m_	Manually collected from published papers	14,669 mutations across 588 proteins	High-quality data collected from the published literature; convenient web interface; a variety of protein stability parameters available; continuously maintained and updated	Limited native protein properties; few sequence data from archaea; small sequence coverage	http://biosig.unimelb.edu.au/thermomutdb	[85]
ProThermDB: Mutant thermal stability data	High-throughput experiments	More than 32,000 proteins and 120,000 thermal stability data	Relatively greater variety of protein sequences; extensive high-quality data from experiments; convenient web interface; continuously maintained	Limited coverage of organisms	https://web.iitm.ac.in/bioinfo2/prothermdb/index.html	[86]
FireProt ^DB^: Mutant thermal stability data	Manually collected from published papers	237 proteins, 13,274 entries	Convenient web interface; high-quality data; multiple sources of annotations;	Limited wild sequence diversity	https://loschmidt.chemi.muni.cz/fireprotdb	[87]
Single-site mutations thermal stability data: *T*_m_, Δ *T*_m_, and Δ *H*	Manually collected from published papers for experimentally measured results	90 wild sequences, 1626 mutant sequences	Various and high-quality thermal stability data; comprehensive quality control	Not easily reusable dataset; not actively maintained; limited number of wild sequences	The Appendix of the article.	[88]

### Large-scale datasets from predictive models

First, Li *et al*. [51] predicted the optimal temperature of 6.5 million enzyme sequences in 4447 enzyme families by constructing several machine learning models, such as linear models, Bayesian ridge, and support vector regression. These models were trained on the growth temperature of microorganisms and the optimal temperature of enzyme sequences in the existing dataset.

Second, Engqvist used the optimal growth temperature (OGT) of 21,498 microorganisms, which contained both bacteria and archaea, spanning a wide range of microorganisms from cryophiles, thermophiles, and hyperthermophiles for predicting the optimum temperature of enzymes [83]. Based on regression models, this dataset enabled the large-scale annotation of protein sequences, which covered approximately 43% of the protein sequences released in UniProt.

### High-quality datasets from published papers and datasets

The prediction of thermal stability data using different algorithms reduces the cost of data collection. However, the quality of the datasets obtained by algorithmic prediction is still not as good as the quality of the manually collected datasets. Therefore, some other researchers constructed the thermal stability dataset of proteins by manually collecting experimental results from the published literature or using high-throughput techniques.

The BRENDA database, a widely used and accessed enzyme annotation database, provides a large amount of hand-curated data on the function and properties of enzymes, including more than 32 million sequences, optimal temperature values for approximately 41,000 enzymes, and temperature stability parameters for approximately 26,000 enzyme sequences [84]. However, it is limited by the high cost of experiments for measuring the thermal stability of enzymes, resulting in a relatively small proportion of labelled sequences in protein families.

The ThermoMutDB missense mutant database contains 14,669 wild-type and mutant thermodynamic data for more than 588 proteins manually collected from the published literature, such as melting temperature ( *T*_m_) and Gibbs free energy [85]. It facilitates downstream tasks by providing database retrieval and usage services through a web interface. By leveraging high-throughput experimental techniques, ProThermDB has more than 32,000 data points on protein thermal stability, such as *T*_m_ and Δ *T*_m_. The database contains more than 120,000 thermodynamic labels of wild types and mutants [86]. FireProt ^DB^ contains the thermal stability data for 740 proteins with more than 25,000 single-point mutants. These data are derived from released datasets and published papers [87].

Pucci *et al*. [88] manually constructed a dataset describing the thermal stability of 31 proteins with single-point mutations, including experimentally determined melting temperature ( *T*_m_), Δ *T*_m_, Δ *G*, and Δ *H*. The principle of data selection was as follows: (1) only single-point mutant sequences, (2) sequences containing protein structures with a resolution of 2.5 Å or less, (3) only data where monomeric proteins were experimentally determined and characterized, (4) protein sequences where the folded and unfolded states of the wild type and mutants were clearly described in the literature, and (5) mutant sequences that caused significant structural changes with temperature ranges beyond 20°C were not included.

The quality of the datasets, which were manually filtered and constructed, was indeed relatively superior. However, the current goal of wild-type–mutant dataset construction led to limited family classes of proteins, hampering the analysis based on evolutionary adaptation. Nonetheless, this problem will be improved with the development of high-throughput technology in the future [89].

## Machine Learning-based Methods and Strategies for Thermal Stability Design of Enzymes

Combining artificial intelligence with high-throughput assays [ 90, 91] , a large number of studies have made important contributions to promote the design of enzyme thermostability. We review three aspects of data-driven strategies for designing enzyme thermal stability, consider recent advances in enzyme thermal stability design, and discuss methods, input data, method reuse, and limitations.

### Prediction of the thermal stability of enzymes

As described in the dataset section, machine learning plays a significant role in dataset augmentation. Furthermore, the ability to accurately predict enzyme thermostability paves the way toward efficient enzyme engineering. In 2009, Ku *et al*. [92] used a statistical method trained on 35 different protein sequences to predict the melting temperature classification directly from protein sequence analysis. The predictor was tested on 75 genomes, including approximately 150,000 proteins, to predict the percentage of high-melting-temperature proteins. The results suggested a correlation between the dipeptide constitution and the melting temperature index. From the perspective of the scale of the training dataset, the very limited amount of data can easily lead to overfitting of the model, which severely limits the generalization ability of the model.

Ten years later, Li *et al*. [51] leveraged the BRENDA database to predict the temperature optima for enzymes based on regression models, but the optimal temperature of enzymes in the training dataset followed a normal distribution. Hence, the optimal temperature data above 85°C was less than 5%, limiting the prediction for thermostable enzymes. Therefore, Gado *et al*. [93] used the ensemble learning and resampling strategy to decrease the mean squared error and increase the overall *R* ^2^ to expand the application range of the predictor. Importantly, in this work, a well-defined accuracy function was applied to narrow the prediction error. The trained model and the code are available on GitHub to facilitate model reuse. The advantages of TOMER are that it is more robust and accurate due to the large training dataset and well-designed resampling strategies.

Recently, a pretrained deep neural network model, DeepET, provided a method for enzyme thermal stability representation [94]. The pretrained model was developed on a dataset of protein sequences with more than 3 million labels of OGT. As a result, the transfer learning models achieved an *R* ^2^ of 0.73 on the test dataset. The attractive advantage of DeepET is that the pretrained model can be reused for the design of downstream tasks and precise prediction with minimum training cost. In addition, the pretrained model can also be used to explore the sequence features that determine the thermal stability factors.

Apart from the regression models for thermal stability prediction, recently, several classification models have shown promising applications for identifying thermostability enzymes, such as iThermo [61] and TMPpred [95]. Shahraki *et al*. [52] presented a sequence-based machine learning model, TaXyl, to classify xylanases from GH10 and GH11 families with different thermal adaptations. This classifier identified three hyperthermophilic xylanases, with maximum activities of 57%–90% at 100°C and 20 min of incubation. Additionally, multilayer perceptron, support vector machine (SVM), and deep learning models were employed to identify thermally stable cellulases [96] and chitinase [97]. Wand *et al*. [53] analyzed protein sequences with kernel principal component analysis and SVM models to identify thermophilic proteins. One of the greatest advantages of these models is that they are user-friendly. Simply taking the protein sequences as input yields a corresponding prediction of thermal stability. On the other hand, these models, trained on specific datasets, are almost only accurate in predicting similar protein sequences. Therefore, sequence families and species need to be considered when using these models. In the high-throughput annotation platform of omics data, these methods can be combined to explore more valuable proteins.

### Prediction of the changes in the stability of mutant proteins

Whether it is functional improvement or design for new enzymes, possessing a thermodynamically stable structure of the enzyme is a basic goal of enzyme engineering [98]. Additionally, the prediction of thermal stability changes upon mutation advances the engineering of enzyme thermal stability design. Giollo *et al*. [99] provided NeEMO for stability changes (ΔΔ *G*) of mutants, which was trained on a large dataset of multiple sequence alignments and protein structures. MAESTROweb is freely accessible on the web server for structure-based protein stability prediction [100]. Another structure-based model, HoTMuSiC, used artificial neural networks with particular activation functions to predict thermal stability changes upon point mutations [101]. Based on a double-checked and refined training dataset, Yang *et al*. [102] trained PON-tstab to predict protein variant stability with 1106 collected features. In this study, several errors and issues with ProTherm [103] entries were proposed, including sequence and structural differences and some errors in stability data. Cao *et al*. [104] developed DeepDDG, which achieved a Pearson correlation coefficient of 0.48–0.56 for three independent test sets based on neural networks. Apart from these tools, several webserver-based tools were used to predict stability changes of mutant proteins, including iStable 2.0 [105], MPTherm-pred [106], and SAAFEC-SEQ [107]. The advantage of the structure-based model, over models trained on sequence-only datasets, improved the model performance by directly embedding structure information corresponding to the function and integrating more expert knowledge into the model.

Certainly, machine learning models provide an efficient way to guide enzyme stability design. Therefore, with the increase in the number of existing prediction models, some studies summarized and assessed these models using benchmark tests [ 108– 111] . At present, these methods have made great breakthroughs and still have great potential for improvement in generalization ability and accuracy.

### Applications of machine learning-driven methods for designing thermostable enzymes

The ability of machine learning to reduce dimensionality enables enzyme engineering to find the sequence composition of targets in a smaller sequence space. Romero *et al*. [112] used Bayesian decision theory, Gaussian process, and the structure-based model for guiding the P450 enzyme thermostability design, which identified sequences that improved the thermal stability by sampling the high-dimensional space of protein sequences, resulting in an average increase of 5.1°C in *T*_50_. For the details of the work, protein structures with at least 50% sequence identity were represented as pairwise interactions between amino acids. However, the disadvantage of this approach is that training and prediction on large datasets is expensive due to the high computational complexity of the algorithm.

Lu *et al*. [64] proposed a self-supervised CNN, MutCompute, to design thermally stable PETase (enzymes that catalyze the hydrolysis of polyethylene terephthalate) by predicting the amino acid positions and types in enzyme sequences that can potentially improve thermal stability. Among all mutants predicted using the model, FAST-PETase with five mutation sites showed the greatest improvement in thermal stability and catalytic efficiency. Compared with wild-type ThermoPETase, the degradation activity of FAST-PETase is increased by 2.4-fold at 40°C and 38-fold at 50°C. The training dataset used by MutCompute contained more than 19,000 protein structures in the Protein Data Bank (PDB) database. The detailed steps of the model are as follows. First, the model creates a microenvironment around the arbitrarily selected central residue within the enzyme and masks all atoms consisting of the central residue, allowing the neural network to predict the type of central residue. Second, the environment contains embedded representation using seven different channels. Third, the trained multilayer CNN network is used to calculate the discrete probability distribution of 20 amino acids at each position in the protein structure. Eventually, if the amino acid types in the corresponding positions are identical, it is considered to be favorable. Otherwise, it is assumed that they need to be optimized. The model architecture that abstracts the complete protein structure into the atomic composition and residue microenvironment provides an enlightening idea for embedding the association between protein structure and function.

Some hybrid methods that combine energy calculation and sequence evolution analysis with machine learning methods have shown strong potential in enzyme thermal stability design [ 113, 114] . The principal disadvantage of hybrid methods, over one predictor, is complicated compiling and environmental setting up for strategy implementation *in silico*. Conversely, the hybrid strategy of combining multiple methods is more universal and feasible in practical applications. The GRAPE strategy screens potentially stable sequences by constructing a single-point mutation library of enzyme molecules. Collaborating with the clustering and greedy algorithm, the *T*_m_ value of DuraPETase is increased by 31°C, and enzymatic PET degradation is increased by more than 300- *fold* at 37°C [115]. Barber-Zucker *et al*. [116] obtained four mutants of versatile peroxidases, exhibiting better resistance in a high-temperature environment relative to the wild type, in which 43 multiple mutations were designed using AlphaFold2 [60], trRosetta [ 117, 118] and PROSS [119]. In this case, the collaboration of evolutionary analysis, protein design models, and accurate structure prediction models was used to proceed with efficient enzyme design.

Pinney *et al*. [120] used a logistic regression model to analyze the embedded enzyme sequences of 1005 enzyme families collected from 5864 bacterial species, resulting in more than 150,000 residues related to the optimal temperature of enzymes. The advantage of this study is that it used a large number of homologous enzyme sequences collected from different protein families and species to illustrate the relationship between temperature adaptability and sequence composition. The output results can be further applied to enzyme design or analyzing the evolutionary strategies of thermal stability for other enzymes. Inspiringly, Singer *et al*. [121] developed a neural network for novel protein sequence generation and protein stability prediction by giving the target secondary structure backbone. The generated thermally stable proteins retained their unfolded and highly thermostable conformations even at temperatures over 99°C. Thus, this approach expands the sequence space for designing thermostable enzymes in a large-scale and efficient manner.

**Figure 4:**
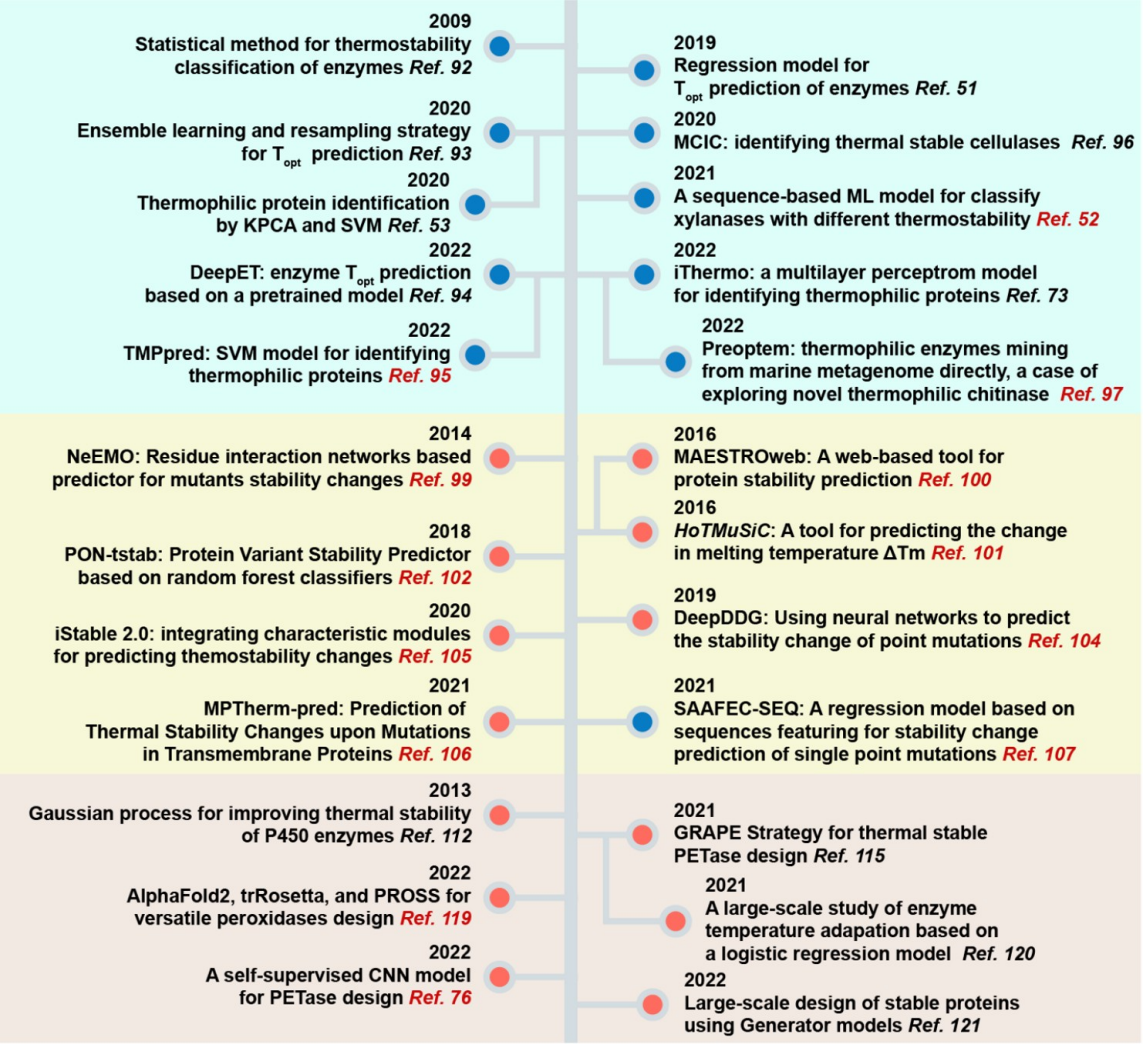
Figure 4 Advanced methods and strategies for enzyme thermal stability design Three aspects of methods are classified by blue, yellow and red blocks. Sequence-only and sequence-structure-based methods are distinguished by blue and red circles, respectively. Red citations indicate that the methods or strategies are freely accessible via web servers.

Although machine learning-driven methods of enzyme thermostability design are limited by data quality and model innovation, current methods and strategies have demonstrated efficient and large-scale design capabilities. It is worth mentioning that the machine learning models are trained on a biased dataset; thus, the applicability of the existing models needs to be noted [122]. Finally, integrating expert knowledge into the machine learning model greatly improves the accuracy and generalization ability [123], especially the computable factors that affect the thermal stability of enzymes and the basic principles of biology [ 18, 32, 124, 125] . Figure 4 summarizes these advanced methods and strategies in a timeline. Most enzyme thermostability prediction methods take only sequence-derived information as input, while thermodynamic stability prediction methods add additional structural information to the models. Moreover, an increasing number of methods are available in the form of online web interfaces for related services. This is a preferable way to use the methods for researchers with no programming background or no experience in using Linux operating systems.

## Challenges for Applying Machine Learning in Enzyme Thermostability Design

The employment of machine learning to cope with the accumulation of biological data brings not only new opportunities for enzyme design but also new challenges for corresponding data management and model training. Here, we present the challenges that may be encountered in guiding enzyme thermal stability design using machine learning.

### Issues and challenges related to datasets

Insufficient, irrelevant, and imbalanced data points hinder the training of machine learning models. When the input label is not constant for the molecular characteristics of the enzyme but varies with the experimental conditions, this type of data can cause the trained model to deviate significantly from the task objective [126]. Therefore, corrective and standardized management should be established to improve the quality of the database.

### Challenges in training models

The training process of the model requires monitoring the relevant metrics to prevent over- and underfitting of the models. When overfitting occurs, the model has been overfitted to the training dataset, which reduces the model’s ability to generalize to unseen data. To prevent overfitting, strategies such as data augmentation, dropout, cross-validation, and multi-model combination are usually helpful [127]. The underfitting situation is generally caused by insufficient training data or high model complexity, which can be countered by appropriately reducing the model complexity.

In recent years, protein language models, a kind of pre-training model, such as ESM-1b and ProtGPT2, have been gaining attention as sequence feature extractors, which can greatly reduce the training cost of models and improve the performance of models for downstream tasks [ 128– 130] . This approach is known as transfer learning and has been used in the past in natural language processing fields, such as bidirectional encoder representations from transformers (BERT) [131].

### Interpretability of machine learning models for enzyme design

As previously mentioned, traditional machine learning methods are the primary choice when facing a new field, benefiting from the interpretability of models and prediction results. In contrast, the inherent black-box modeling framework of deep learning leads to weak interpretability and challenges the complex design of enzymes. Therefore, interpretable neural networks should be given more attention, which is crucial for the field of enzyme design [132].

### Evaluation and utilization of existing models

A mature machine learning model must be subject to a complex evaluation process that includes statistical evaluation, biochemical experiments, and computational simulations. For statistical evaluation, the choice of metrics is critical. Both biochemical experiments and fine-grained computational simulations can be relatively costly. Therefore, high-throughput techniques and sophisticated experimental design can largely improve experimental efficiency.

A large number of models are available for the design of enzymes with thermal stability. However, the use of these models is hampered by the fact that most of these methods require skills to use the Linux operating system and some programming languages. Furthermore, the differences in the interfaces of the methods and the required compilation environment dependencies make it difficult for researchers to evaluate and select the best one among different methods. Nevertheless, similar platforms exist in other areas of life science that provide informative insights into this issue. For example, critical assessment of protein structure prediction (CASP) and critical assessment of protein function annotation algorithms (CAFA) provide benchmarks for evaluating computational methods of protein structure and function, respectively [ 133, 134] .

## Conclusion and Future Direction

This review summarizes recent advances in machine learning–based methods for designing the thermal stability of enzymes. Among the introduced related datasets, a large amount of data was obtained by model prediction and manually collected from published literature. These available and accessible datasets provide a rich material basis for the training of machine learning models. After decades of development, models using different data, such as sequences, structures, and thermodynamics data, have contributed to the design of the thermal stability of enzymes. In addition, machine learning-based methods provide a sustained and efficient way of exploring the sequence- or structure-fitness landscape of proteins [ 135, 136] . We also presented some challenges in applying machine learning for designing enzyme thermostability.

The revolutionary breakthrough of AlphaFold2, which has achieved high accuracy and efficiency in protein structure prediction, promises to be the fourth most efficient way to obtain protein structures [ 60, 137] . AlphaFold2 recently open-sourced more than 200 million protein structures, bridging the data gap between protein sequence and structure and enabling the incorporation of protein structure information into machine learning models [ 138, 139] . This breakthrough laid the foundation for the genome-wide applications of protein structure-based artificial intelligence models [140]. Diverse successful applications that provide feasible solutions to the design of the thermal stability of enzymes are available. The unified and standardized platform for managing and comparing data resources and models is highly conducive to large-scale utilization and promotion.

Approximately 20 years ago, researchers leveraged whole-genome sequencing technology to obtain the genetic code for encoding life [ 141, 142] . We now stand at a critical point between the explosive generation of big biological data and the booming field of machine learning. A large number of techniques have enabled us to completely understand and decode how nature creates life. Harnessing machine learning models to observe the flow of information about life provides us with a powerful grip to find the patterns behind it. We look forward to the next groundbreaking advances in the field of enzyme design achieved by machine learning.

## Supporting information

496Table
