# Supplementary material for: Data-driven strategies for the computational design of enzyme thermal stability: trends, perspectives, and prospects: Data-driven strategies for enzyme thermostability design
Source: Acta Biochim Biophys Sin (Shanghai). 2023 Mar 16;55(3):343–55. doi: 10.3724/abbs.2023033 (PMC10160227; doi:10.3724/abbs.2023033)
Supplement: 496Table [file 496Table.pdf]

Table 1. Enzyme thermal stability datasets

Dataset description	Collection method	Dataset scale	Advantages	Disadvantages	Availability	Reference
Tome: optimal temperature of enzyme	Predicted by linear models, Bayesian ridge, and support vector regression	4,447 enzyme families, 6,500,000 sequences	Large-scale protein sequences and family diversity facilitating training of machine learning models; easy-to-access public dataset	Insufficient accuracy of prediction methods; low accuracy of data on the optimum temperature of enzymes in the extreme temperature range	https://zenodo.org/record/2539114	[51]
The optimal growth temperature of bacteria and the optimal temperature of enzyme	Correlation analysis between enzyme temperature optima and the organism growth temperatures	21,498 OGT of bacteria	UniProt sequences covered by 43%; easy-to-access public dataset; covering a variety of temperature-adapted bacteria and archaea.	Stricter quality control needed; not actively maintained	https://doi.org/10.5281/zenodo.1175608	[83]
BRENDA: The optimal temperature of enzymes	Published papers in PubMed	32,000,000 sequences with 41,000 optimal temperature labels	High-quality data collected from the published literature; actively maintained; convenient web interface	Relatively small proportion of temperature-labeled sequences in protein families; no obviously experimental conditions	https://www.brenda-enzymes.org/	[84]
ThermoMutDB: Environment, $\Delta\Delta G$, ΔT_m	Manually collected from published papers	14,669 mutations across 588 proteins	High-quality data collected from the published literature; convenient web interface; a variety of protein stability parameters available; continuously maintained and updated	Limited native protein properties; few sequence data from archaea; small sequence coverage	http://biosig.unimelb.edu.au/thermomutdb	[85]

ProThermDB: Mutant thermal stability data	High-throughput experiments	More than 32,000 proteins and 120,000 thermal stability data	Relatively greater variety of protein sequences; extensive high-quality data from experiments; convenient web interface; continuously maintained	Limited coverage of organisms;	https://web.iitm.ac.in/bioinfo2/prothermdb/index.html	[86]
FireProt^{DB}: Mutant thermal stability data	Manually collected from published papers	237 proteins, 13,274 entries	Convenient web interface; high-quality data; multiple sources of annotations;	Limited wild sequence diversity;	https://loschmidt.chemi.muni.cz/fireprotdb	[87]
Single-site mutations thermal stability data: Tm, ΔT_m, and ΔH	Manually collected from published papers for experimentally measured results	90 wild sequences, 1,626 mutant sequences	Various and high-quality thermal stability data; comprehensive quality control	Not easily reusable dataset; not actively maintained; limited number of wild sequences	The Appendix of the article.	[88]
